# The Importance of Hemoptysis as a Sign of Pulmonary Phthisis

**Published:** 1857-05

**Authors:** Edward De Lamare


*The Importance of Hemoptysis as a Sign of Pulmonary Phthisis.* Read before the Academy of Sciences, by Dr. EDWARD DE LAMARE. (From the *Gazette Medicale.*)

All pathologists agree in regarding hemoptysis, or spitting of blood, as a symptom of the invasion of the pulmonary parenchyma by tubercles, which are deposited, not as has been advanced, in the pulmonary vesicles, but in the inter-areolar cellular tissue, not only of the lung, but also of the other organs. Although hemoptysis may be very frequently a sign of phthisis, there are cases, rare to be sure, yet undoubted, of idiopathic hemoptysis, which are not allied to any affection of the air-passages. M. Louis, in his treatise upon pulmonary phthisis, says that the cases of idiopathic hemoptysis are to those which depend upon phthisis as 1 is to 2,400, which is the same as to say that when an individual spits blood, there is little doubt that he is tuberculous. The experience resulting from my own observations, leads me to lay down this relation as 1 to 66; which still making phthisis the most usual condition, yet greatly enlarges the field of exceptions; for 66 : 2,400 : : 1 : 36⅓. Consequently, still maintaining the gravity of the prognosis after hemoptysis, my calculation, made from patients submitted to my observation, is 36 to 37 less unfavorable than that given by M. Louis.

In making these statistics I have rejected all doubtful cases, and have only admitted those as idiopathic hemoptysies which, dating back fifteen years, were neither preceded nor followed by a cough, even of a few days, nor by loss of flesh, nor loss of strength, in persons who had no hereditary tendency to phthisis, who were not subject to colds, and whose lungs, examined by myself fifteen years after the accident, by means of auscultation, were found perfectly healthy.

There is a difference between men and women as regards idiopathic hemoptysis. It is more frequent in women whose menstrual evacuations often, either suppressed or diminished, tend to be replaced by supplementary hæmorrhages; thus, while for the two sexes united the relation is as 1 to 66, it is as 1 to 132 for men, and as 1 to 33 for women.

I do not designate by hemoptysis, a few streaks or spots of blood which one may eject with the sputa without being consumptive, but the expectoration of a considerable quantity of blood.

Independently of hemoptysies purely idiopathic, there are individuals who spit blood under the influence of a tuberculous disease, but in whom, however, this disease remains almost stationary, and in the latent state, while they are in good hygienic conditions. The individuals, it may be stated, give birth to children who ordinarily appear phthisical before they reach the age of their parents. It is evident that in this hereditary transmission, the intensity of the disease had increased a step.

As to the frequency of hemoptysies in pulmonary phthisis, the observation of facts prevent me from subscribing to the statement made in M. Louis work, that one-half of consumptives have hemoptysis, while the other half have none. The cases accompanied by spitting of blood, are more frequent than those in which this sign is absent. The relation is as 75 to 55. Finally, while idiopathic hemoptysis is more frequent in the female than in man, it is the contrary as to hemoptysis due to pulmonary phthisis. For instance, from 130 consumptives submitted to my observation, of which 65 were men and 65 women, 75 had spat blood, of which 45 were men and 35 women.—*Amer. Medical Monthly.*

